# Xiaoai Jiedu recipe promotes NLRP3 inflammasome activation by alleviating PINK1-mediated mitophagy in hepatocellular carcinoma

**DOI:** 10.3389/fonc.2026.1985999

**Published:** 2026-10-27

**Authors:** Yuan-yuan Wu, Hui-ming Zhu, Zi-yu Lu, Zhuo-hui Tang, Wen-ting Li, Mian-hua Wu

**Affiliations:** 1 First Clinical Medical College, Nanjing University of Chinese Medicine, Nanjing, China; 2 The Affiliated Hospital of Nanjing University of Chinese Medicine, Jiangsu Province Hospital of Chinese Medicine, Nanjing, China; 3 Jiangsu Collaborative Innovation Center of Traditional Chinese Medicine in Prevention and Treatment of Tumor, Nanjing, China

**Keywords:** hepatocellular carcinoma (HCC), mitophagy, NLRP3 inflammasome, traditional Chinese medicine (TCM), Xiaoai Jiedu recipe (XJR)

## Abstract

**Background:**

The Xiaoai Jiedu recipe (XJR), a traditional Chinese medicinal formula, has been applied clinically for liver cancer management. XJR exhibits promising anti-tumor potential and may offer a novel strategy for oncological intervention. This study was designed to investigate the molecular mechanisms by which XJR exerts its pharmacological effects against hepatocellular carcinoma (HCC).

**Methods:**

*In vivo* assays were conducted employing an HCC xenograft mouse model, and HepG2 cells were employed *in vitro*. ELISA, immunohistochemistry (IHC), immunofluorescence (IF), RT-PCR and western blot were performed to detect PTEN-induced putative kinase 1 (PINK1)-mediated mitophagy and NLRP3 (NOD-like receptor protein 3) inflammasome activation. The cellular mitochondrial reactive oxygen species (ROS) levels were tested with DCFH-DA and MitoSOX. Transmission electron microscopy (TEM) and JC-1 were utilized to observe the mitochondrial morphology and function, as well as mitochondrial membrane potential (MMP) in the process of XJR intervention for HCC.

**Results:**

XJR prominently promoted pyroptosis by upregulating the related inflammatory cytokines levels in serum and HepG2 cells. The key targets include PINK1, parkin, p62, LC3B, TOMM20, COX IV, NLRP3, ASC, GSDMD, caspase1, IL-1β and IL-18 were observed. XJR disrupted PINK1-mediated mitophagy, which in turn increased mitochondrial ROS levels and promoted NLRP3 inflammasome assembly both *in vitro* and *in vivo*. Meanwhile, functional assays using pathway-specific inhibitors and siRNA-mediated knockdown further demonstrated that XJR suppresses mitophagy and enhances NLRP3 inflammasome activation via inhibition of the PINK1/parkin signaling pathway.

**Conclusions:**

The preliminary results of the present study suggested that XJR may alleviate PINK1-mediated mitophagy and motivate NLRP3 signaling pathway to subsequently exert anti-tumor effects in HCC.

## 1 Introduction

As indicated by the Global Cancer Statistics of 2022, liver cancer is reported as the third major cause of cancer-related mortality worldwide (1). Hepatocellular carcinoma (HCC) accounts for approximately 90% of all primary liver cancer instances. Owing to the absence of distinct clinical symptoms in its initial phase, the disease is frequently diagnosed at an advanced stage (2). HCC is characterized by high recurrence and mortality rates, creating an urgent demand to identify effective and reliable therapeutic targets. Current clinical strategies for improving patient survival include chemotherapy, radiotherapy, immunotherapy, liver transplantation, surgical resection and ablation, and molecularly targeted drugs. Nevertheless, conventional therapeutic approaches are severely limited by prolonged disease latency, multidrug resistance, frequent tumor recurrence, and substantial adverse drug reactions (3, 4). Therefore, clarifying the mechanisms of restraining HCC is critical for developing new therapeutic strategies.

Mitophagy is a selective autophagic process essential for maintaining cellular homeostasis via the targeted recognition and removal of damaged mitochondria (5). Extensive research indicates that mitophagy exhibits a dual function in HCC, simultaneously promoting and suppressing its initiation and progression (6). As a transducer of metabolism-related risk signaling, the NOD-like receptor protein 3 (NLRP3) inflammasome is essential for the production of inflammatory cytokines and participates in the regulation of mitochondrial function, serving as a core mediator driving HCC progression (7). Numerous previous studies have illustrated that the PINK1/parkin pathway serves as a critical signaling axis that participates in the regulation of mitophagy (8). Through phosphorylation and ubiquitination, PINK1 is able to sense mitochondrial injury and trigger parkin activation, a process that is closely associated with HCC progression and the activation of the NLRP3 inflammasome (9). The suppression of mitophagy has been shown to enhance the activation of the NLRP3 inflammasome activation (10). Additionally, research findings indicate that mitophagy dependent on PINK1/parkin may exert a negative impact on its anti-cancer effects in HCC (11–13). Targeted deletion of the mitophagy-associated proteins PINK1 and parkin augments NLRP3 inflammasome activation upon exposure to diverse NLRP3 agonists (14). Conversely, mitophagy induction substantially reduces the cleavage of pro-caspase-1 and pro-IL-1β, reflecting suppressed inflammasome activity (15). What’s more, mitochondria-derived reactive oxygen species (ROS) act as a key upstream activator of the NLRP3 inflammasome (16), which also represents a downstream signal of mitochondrial dysfunction. Additional investigations are required to clarify the relevance of mtROS to the crosstalk between mitophagy and NLRP3 inflammasome activation.

As a precious cultural heritage of the Chinese nation, Traditional Chinese Medicine (TCM) has been refined and passed down over thousands of years and maintains an irreplaceable role in contemporary healthcare. Particularly in oncology, TCM herbs and their active ingredients demonstrate notable anti-HCC potential, attributed to their unique advantages of multi-component, multi-target, and multi-pathway regulatory properties (17, 18). With the continuous in-depth research on mitophagy, growing interest has focused on the regulatory roles of TCM in HCC (19). Xiaoai Jiedu Recipe (XJR), a traditional Chinese medicinal formula established by Professor Zhou Zhongying, consists of seven herbal components (**Table 1**). XJR suppresses cell viability and promotes apoptosis in HCC cells through the AKT/mTOR signaling pathway (20), and attenuates HCC proliferation via the miR-200b-3p/Notch1 axis. and decreases HCC proliferation via the miR-200b-3p/Notch1 axis (21). Additionally, XJR has been shown to possess distinctive antitumor effects and reduce the toxicity of chemotherapeutic agents, thereby significantly improving the quality of life in patients (22). This study was designed to investigate the molecular processes through which XJR exerts its effects on HCC, with a particular focus on mitophagy and NLRP3 inflammasome. This study verified that XJR modulates PINK1-mediated mitophagy and the NLRP3 signaling pathway in its anti-HCC actions.

**Table 1:** Formulation of Xiaoai Jiedu recipe (XJR).

Pinyin name	English name	Latin pharmaceutical name	Doses	Origin
Baihuasheshecao	Spreading Hedyotis Herb	Herba Hedyotidis	20g	The dried whole herb of *Scleromitrion diffusum* (Willd.) R.J.Wang.
Shancigu	Appendiculate Cremastra Pseudobulb	Cremastrae Pseudobulbus Pleiones Pseudobulbus	10g	The dried pseudobulb of *Cremastra appendiculata* (D. Don) Makino.
Bayuezha	Fruit of Fiveleaf Akebia	Fructus Akebiae	12g	The Dried fruit of *Akebia quinata* (Thunb. ex Houtt.) Decne.
Jiangcan	Stiff Silkworm	Bombyx Batryticatus	10g	The dried body of the larvae of *Bombyx mori* L that has been killed due to infection (or inoculation) of the fungus Beauveria bassiana (Bals.) Vuill.
Wugong	Centipede	Scolopendra	3g	The dried body of *Scolopendra subspinipes* mutilans L. Koch
Taizisheng	Heterophylly Falsestarwort Root	Pseudostellariae Radix	15g	The dried tuberous root of *Pseudostellaria heterophylla* (Miq.) Pax et Hoffm.
Maidong	Dwarf Lilyturf Root Tuber	Ophiopogonis Radix	12g	The dried tuberous root of *Ophiopogon japonicas* (Thunb.) Ker-Gawl.

## 2 Methods

### 2.1 Materials and reagents

XJR was provided by Anhui Bozhou Traditional Chinese and Western Medicine Co., Ltd. and Suzhou Tianling Traditional Chinese Medicine Decoction Pieces Co., Ltd. (Anhui, China). Detailed analytical data supporting the chemical characterization of the herbal formulation have been reported in our previous published study (23). All plant denominations were validated via the database of http://www.worldfloraonline.org, with the website accessed and referenced on October 3, 2024. The herbal materials employed in the study were as follows: Spreading Hedyotis Herb (Batch No.: 2021082201), Appendiculate Cremastra Pseudobulb (Batch No.: CG08001), Fruit of Fiveleaf Akebia (Batch No.: CR02801), Stiff Silkworm (Batch No.: 2022033103), Centipede (Batch No.: CD03101), Heterophylly Falsestarwort Root (Batch No.: 2022022301), and Dwarf Lilyturf Root Tuber (Batch No.: CG01601).

In addition, we have examined the UPLC-Q-TOF-MS/MS of chemical components of XJR and placed the results in the **Supplementary Figure 1** and **Supplementary Figure 2**. The analysis was performed on a Thermo Fisher Scientific Q Exactive Orbitrap mass spectrometer coupled with a Dionex Ultimate 3000 RSLCnano system. Chromatographic separation was carried out using an Acclaim RSLC 120 C18 column (2×150 mm, 2.2μm). The mass spectrometry scan was operated in both positive and negative ion modes with a mass range of m/z 50-1500. The Full MS/dd-MS² scanning mode was adopted, and the resolution of the full-scan MS and MS/MS were set to 140,000 and 17,500, respectively. The detailed ESI source parameters were strictly controlled as follows: sheath gas flow rate of 40, auxiliary gas flow rate of 15, sweep gas flow rate of 0, spray voltage of 3.2 kV (positive mode) and 2.8 kV (negative mode), capillary temperature of 320 °C, S-lens RF level of 55, and auxiliary gas heater temperature of 350 °C. For liquid chromatographic elution, the mobile phase consisted of 0.1% formic acid aqueous solution (phase A) and acetonitrile (phase B) at a flow rate of 0.2 mL/min. The gradient elution procedure was programmed as follows: 0–30 min, 10%-18% B; 30–44 min, 18%-40% B; 44–46 min, 40%-60% B; 46–50 min, 60%-100% B. For chemical component identification and data analysis, we first systematically reviewed and summarized the reported phytochemical constituents of traditional Chinese medicines related to XJR to construct a local chemical component database containing compound names, molecular formulas, and structural information. Meanwhile, public online databases including Massbank, PubChem, ChemSpider, and MzCloud were integrated to supplement and optimize the compound database for accurate component matching. Based on the established UPLC-Q-TOF-MS/MS detection method and standardized database screening strategy, a total of 93 chemical constituents were successfully identified and characterized in XJR decoction. The structural information, mass spectral fragmentation rules, and relative content distribution of all identified constituents have been supplemented in the revised Results section and attached **Supplementary Figures** / **Supplementary Tables**.

The prestained protein ladder (WJ103) was obtained from Yamei Biotech (Shanghai, China). Antibodies against parkin (2132), p62 (5114), LC3B (2775), Caspase1 (83383), Cleaved Caspase-1(89332; 4199), IL-1β (12242), IL-18 (72254), Caspase3 (9662), GAPDH (5174) and β-actin (4970) were purchased from Cell Signaling Technology (Danvers, United States). Antibody against PINK1(ab216144), p62 (ab91526), LC3B (ab192890), TOMM20 (ab186735), COX IV (ab16056), NLRP3 (ab270449, ab263899), ASC (ab309497), GSDMD (ab219800), Caspase1 (ab138483), IL-1β (ab216995, ab283818), and Ki67 (ab16667) were purchased from Abcam (Cambridge, UK). The PINK1(HA723021), parkin (ET1702-60), NLRP3 (ER1706-72), GSDMD-N (ER1901-37), Caspase1 (R1510-23) and IL-18 (72254) antibodies were obtained from HUABIO (Zhejiang, China). A complete list of all antibodies is provided in **Supplementary Table 1**.

### 2.2 Medicated serum preparation

Twenty-four healthy adult male Sprague-Dawley rats aged 6–8 weeks and weighing 220–250 g were supplied by Qing Long Shan Animal Breeding Center (Nanjing, China). Rats in the intervention groups were intragastrically administered a dose of XJR at 9 g/kg body weight once daily. An equivalent volume of normal saline was administered to the control group via the same intragastric administration route. Blood samples were harvested subsequent to the eighth dosing and subjected to centrifugation to isolate the supernatant. The collected serum was inactivated by heating at 56 °C for 30 min, sterilized by filtration through 0.22 μm filters. For subsequent cell experiments, the serum was diluted to 15% concentration with serum-free culture medium (24).

### 2.3 Animals

Healthy athymic BALB/c-nu male mice (4–6 weeks; 20 ± 2 g) were obtained from Qing Long Shan Dong Wu Fan Zhi Chang (Nanjing, China) and raised under specific pathogen free conditions with free access to water and food. All animals were housed under specific pathogen-free (SPF) conditions with a 12 h light-dark cycle, constant temperature (22 ± 2 °C) and relative humidity (50% ± 10%). Mice had unrestricted access to sterilized standard rodent chow and autoclaved drinking water throughout the experimental period. H22 cells (5×10^6^ per mouse) were subcutaneously injected into the flank of mice to establish the HCC xenograft model (21). All *in vivo* experiments strictly followed institutional animal ethical guidelines. Mice were randomly assigned to each group using a random number table to reduce selection bias, with six mice per group. The length and width of subcutaneous tumors were measured every 3 days using a vernier caliper, and tumor volume was calculated as follows: tumor volume = length × width²/2. Predefined humane endpoints were applied to minimize animal suffering. Mice were monitored daily to assess tumor growth. After the tumor diameter attained 5–6 mm, the animals were randomly assigned to four groups (n=6): the control group treated with an equivalent volume of normal saline, and three experimental groups administered XJR at low (XJR-Low, 1 g/kg body weight/day), medium (XJR-Middle, 3 g/kg body weight/day), and high (XJR-High, 9 g/kg body weight/day) doses, respectively (21). All mice received once-daily intragastric administration for 14 consecutive days. At 24 h after the final dose, mice were euthanized, and tumor tissues and blood samples were harvested for further analysis. Prior to blood sampling, animals were anesthetized using isoflurane (induction 2-3%, maintenance 1.5-2%). Blood was collected from the orbital arteriovenous plexus, after which euthanasia was performed via cervical dislocation. The experimental protocol was approved by the Animal Care and Use Committee of Nanjing University of Chinese Medicine. (Approval Number: 202403A061).

### 2.4 Cell lines and cultures

HCC lines (HepG2 and H22 cells) were purchased from the Chinese Academy of Sciences Shanghai Branch Cell Bank (Shanghai, China). HepG2 cells were pretreated with NLRP3 inhibitor (MCC950) (10μM; S7809, Selleck, USA) and autophagy inhibitor 3-methyladenine (3-MA) (5 mM; HY-19312, MedChem Express, China) as a reference control, respectively (25, 26). For cell culture, commercial fetal bovine serum (FBS) was heat-inactivated to eliminate complement components and sterile-filtered prior to addition to complete culture medium. Upon reaching approximately 50% confluence, transfection was performed using lipofectamine 3000 (Invitrogen) with either NC-siRNA (sc-37007) or PINK1-siRNA (sc-44598) obtained from Santa Cruz (Heidelberg, Germany). In brief, a total of 8 µL Lipofectamine 3000 was diluted into 100 µL Opti-MEM medium and equilibrated at room temperature. In parallel, 8 µL siRNA (final concentration 100 nM) was diluted in a separate 100 µL Opti-MEM. After a 5-min incubation, the two mixtures were gently combined and incubated at room temperature for another 15 min to facilitate formation of transfection complexes. The resulting transfection mixture was added dropwise onto the cells, and cells were incubated at 37 °C with 5% CO_2_ for 8 h. After transfection, cells were cultured for 48 h prior to subsequent treatments as described above.

### 2.5 Histological and immunohistochemical analysis

Tumor specimens were fixed in 4% paraformaldehyde, dehydrated through a graded ethanol series, embedded in paraffin, and sectioned into thin slices. The sections were then subjected to H&E staining, and histopathological changes in tumor tissues were examined under a light microscope. Immunohistochemistry (IHC) (Sangon Biotech, Shanghai, China) was utilized to assess the degree of pathological changes (Ki67, Caspase 3) and evaluate the effects of XJR on the levels of PINK1-mediated mitophagy and NLRP3 signaling pathway markers (PINK1, parkin, p62, LC3B, NLRP3, ASC, GSDMD, caspase1, IL-1β and IL-18) in tumor tissues.

### 2.6 Enzyme-linked immunosorbent assay

A supernatant was extracted after the blood was centrifuged at 3500 r/min for 20 min, furthermore, a certain amount of PBS was added into the serum of mice or HepG2 cells, homogenized fully and the sample was centrifuged. Levels of IL-1β (YFXEM00028, mouse), IL-18 (YFXEM00573, mouse), IL-1β (YFXEH00259, human) and IL-18 (YFXEH00250, human) in supernatant of serum and cells were measured using ELISA kits (YFXBIO, China), following the manufacturer’s instructions.

### 2.7 LDH release assay

LDH release was detected via an LDH cytotoxicity assay kit (C0017, Beyotime, China). HepG2 cells were plated in 96-well plates at 1 × 10^4^ cells/well and subjected to XJR treatment for 24 h. LDH release reagent was supplemented 1 h before sample detection. After centrifugation, supernatants were collected and incubated with LDH working solution away from light for 30 min, and absorbance at 490 nm was recorded using a microplate reader.

### 2.8 Transmission electron microscopy

The ultrastructure of mitochondria and autophagic vesicles of tumor tissues and HepG2 cells were observed by transmission electron microscopy (JEOL JEM-1400Flash). Following euthanasia, tumor tissues were rapidly dissected into 1mm×1mm×1mm blocks, fully immersed in the transmission electron microscopy solution and stored at 4°C overnight. Besides, cells were harvested and fixed in 2.5% glutaraldehyde at 4 °C for 2 hours. Samples were dehydrated using a graded ethanol series followed by pure acetone. After incubation with 1% osmium tetroxide at room temperature for 2 h, the specimens were embedded in epoxy resin (Embed 812, Electron Microscopy Science, Perth, Australia). Ultrathin sections were fabricated and stained with lead citrate and 2% uranyl acetate dissolved in ethanol for further observation.

### 2.9 Measurement of mitochondrial reactive oxygen species

The fluorescent probe 2’,7’-dichlorofluorescein diacetate (DCFH-DA; S0039, Beyotime) and MitoSOX Red Mitochondrial Superoxide Indicator (M36008, Invitrogen) were employed to visualize cellular reactive oxygen species. Following treatment, cells were incubated with 10 mM DCFH-DA, 1 μM MitoSOX Red, and Hoechst 33342 at 37 °C in the dark for 25 minutes. A fluorescence microscope (ZEISS, Germany) was employed to record the fluorescence signals of intracellular reactive oxygen species.

### 2.10 Measurement of mitochondrial membrane potential (Δψm)

A commercial MMP detection kit (JC-1; C2003S, Beyotime) was utilized to determine mitochondrial membrane potential (MMP) following the protocol. Post the indicated treatment, cells were incubated with 1 mL of JC-1 staining working solution per well for 20 min. Hoechst staining was applied for nuclear counterstaining, and fluorescence signals were detected using a fluorescence microscope.

### 2.11 Immunofluorescence

After the designated treatment, cells were fixed for 20 minutes in ice-cold 4% paraformaldehyde and permeabilized with 0.5% Triton X-100 for a duration of 5 minutes. The samples were incubated with 1% BSA at room temperature for 1 hour and incubated with rabbit-derived antibodies and FITC-conjugated secondary antibodies. The apoptosis of tumor tissues and cells was expressed according to the TUNEL (Beyotime, C1086). DAPI (Southern Biotech, United States) was employed for nuclear counterstaining. The images were obtained by an inverted fluorescence microscope under 400×magnification.

### 2.12 Real-time quantitative polymerase chain reaction

Total RNA was extracted from tumor tissue and cells and real-time quantitative polymerase chain reaction (RT-qPCR) was performed according to protocol of manufacturer’s instructions. Level of β-actin messenger RNA (mRNA) was set as an internal control (B662302-0001, B662102-0001; Sangon Biotech, Shanghai, China). Primers for *PINK1*, *parkin*, *p62*, *LC3B*, *TOMM20*, *COX IV*, *NLRP3*, *ASC*, *GSDMD*, *caspase1*, *IL-1β* and *IL-18* were designed and used. Quantitative primers for amplification were showed as described below (**Table 2**).

**Table 2:** Primers for quantitative polymerase chain reaction.

Gene	Forward	Reverse
*PINK1(Mouse)*	CCACCTTTCCCTTTGCCATC	TGCTTGGGACCATCTCTGGA
*parkin(Mouse)*	ACCTGCAAACAAGCAACCCT	CTACCGACGTGTCCTTGTCT
*LC3B(Mouse)*	CCGAACCGCAGACGCAT	CTGCACCTCCTTACAGCGG
*p62(Mouse)*	AGTCAGCTTCTGCTCCACCA	TTGTCTTCTGTGCCTGTGCT
*TOMM20(Mouse)*	TACTGCATCTACTTCGACCGC	CCAGCTCTCTCCTTAGCAAGC
*COX IV(Mouse)*	TCATTGGCTTCACTGCGCTCGT	TCCAGCATTCGCTTGGTCTGCA
*NLRP3(Mouse)*	ATTGCTGTGTGTGGGACTGAA	ATCCTGACAACACGCGGAT
*ASC(Mouse)*	CTTACAGGAGCTGGCTGAGCA	GACCCTGGCAATGAGTGCTTG
*GSDMD(Mouse)*	CCATCGGCCTTTGAGAAAGTG	ACACATGAATAACGGGGTTTCC
*caspase-1(Mouse)*	TGCTTTCTGCTCTTCAACACCA	CCAAGTCACAAGACCAGGCATAT
*IL-1β(Mouse)*	TGGTGTGTGACGTTCCCATT	TGTCGTTGCTTGGTTCTCCT
*IL-18(Mouse)*	GCGTCAACTTCAAGGAAATGATGT	TGTCAACGAAGAGAACTTGGTCAT
*PINK1(Human)*	CTCCAGCGAAGCCATCTTGA	CGGAGAACCCGGATGATGTT
*parkin(Human)*	ACCAGCAGTATGGTGCAGAG	CGCTTCTTTACATTCCCGGC
*LC3B(Human)*	AACATGAGCGAGTTGGTCAAGAT	TCACCAGCAGGAAGAAGGC
*p62(Human)*	AATGGGCCTGTGGTAGGAAC	CTGTGCGAGAAGCCCTCAGA
*TOMM20(Human)*	CAGCGTTGTAGAGAAGATGGT	AGGTAACTTGGAAAGCCCAGC
*COX IV(Human)*	CAGGGTATTTAGCCTAGTTGGC	GCCGATCCATATAAGCTGGGA
*NLRP3(Human)*	GATCTTCGCTGCGATCAACA	GGGATTCGAAACACGTGCATTA
*ASC(Human)*	GGATGCTCTGTACGGGAAGG	AGGCTGGTGTGAAACTGAAGAG
*GSDMD(Human)*	GTGTGTCAACCTGTCTATCAAGG	CATGGCATCGTAGAAGTGGAAG
*caspase-1(Human)*	GCCTGTTCCTGTGATGTGGA	ACTCTTTCAGTGGTGGGCAT
*IL-1β(Human)*	CCAGGGACAGGATATGGAGCA	TTCAACACGCAGGACAGGTACAG
*IL-18(Human)*	GACCAAGTTCTCTTCATTGACCAA	AGATAGTTACAGCCATACCTCTAGG
Gene sequences (5’ to 3’)		

### 2.13 Western blot

Cell lysates and tumor tissues were homogenized on ice and centrifuged to collect supernatant. Protein extracts were separated by sodium dodecyl sulfate-polyacrylamide gel electrophoresis (SDS-PAGE) and transferred onto PVDF membranes. The membranes were then blocked with 5% skimmed milk and incubated with primary antibodies overnight at 4 °C. This was followed by treatment with HRP-conjugated secondary antibodies (PR30011, Proteintech Group) and visualization with a ChemiDoc MP imaging system (Bio-Rad, Hercules, CA, USA).

### 2.14 Statistical analysis

The experimental datasets and relevant quantitative diagrams were generated via GraphPad Prism 9.0. All data were expressed as mean ± standard deviation (SD) from at least three independent experiments. Prior to statistical analysis, Shapiro-Wilk normality test was conducted to verify the prerequisites for parametric tests. Two-group comparisons were carried out using unpaired Student’s t-test, while multiple-group comparisons adopted one-way ANOVA followed by Tukey *post hoc* test. Differences with P < 0.05 were regarded as statistically significant. Corresponding P-values were annotated within figure legends.

## 3 Results

### 3.1 *In vivo* antitumor efficiency of XJR

A subcutaneous HCC xenograft model was constructed by subcutaneous inoculation of H22 cells into BALB/c-nu mice to evaluate the anti-tumor effect of XJR *in vivo.* The dynamic tumor growth curves, terminal tumor weights, and representative photographs of excised tumors are presented in **Supplementary Figure 3**. H&E staining was performed to evaluate histopathological alterations in tumor tissues, aiming to verify the therapeutic efficacy of XJR against HCC. As illustrated in **Figure 1**, XJR-administered mice showed notably more serious structural damage in their tumor tissues, such as irregularly widened intercellular spaces, extensive necrosis, and nuclear condensation and pyknosis. Meanwhile, Ki67, a marker of cell proliferation, was applied to assess the effect of XJR on tumor growth. The results revealed that XJR treatment markedly reduced Ki-67 expression. In addition, the TUNEL assay demonstrated a prominent dose-dependent increase in the apoptotic rate of HCC cells upon XJR treatment was used to evaluate the effect of XJR on tumor growth. Immunohistochemical staining further validated this observation: XJR treatment significantly increased apoptotic markers, as reflected by elevated Caspase-3 expression. These findings indicate that XJR exerts a notable inhibitory effect on tumor growth and invasion in HCC.

**Figure 1:**
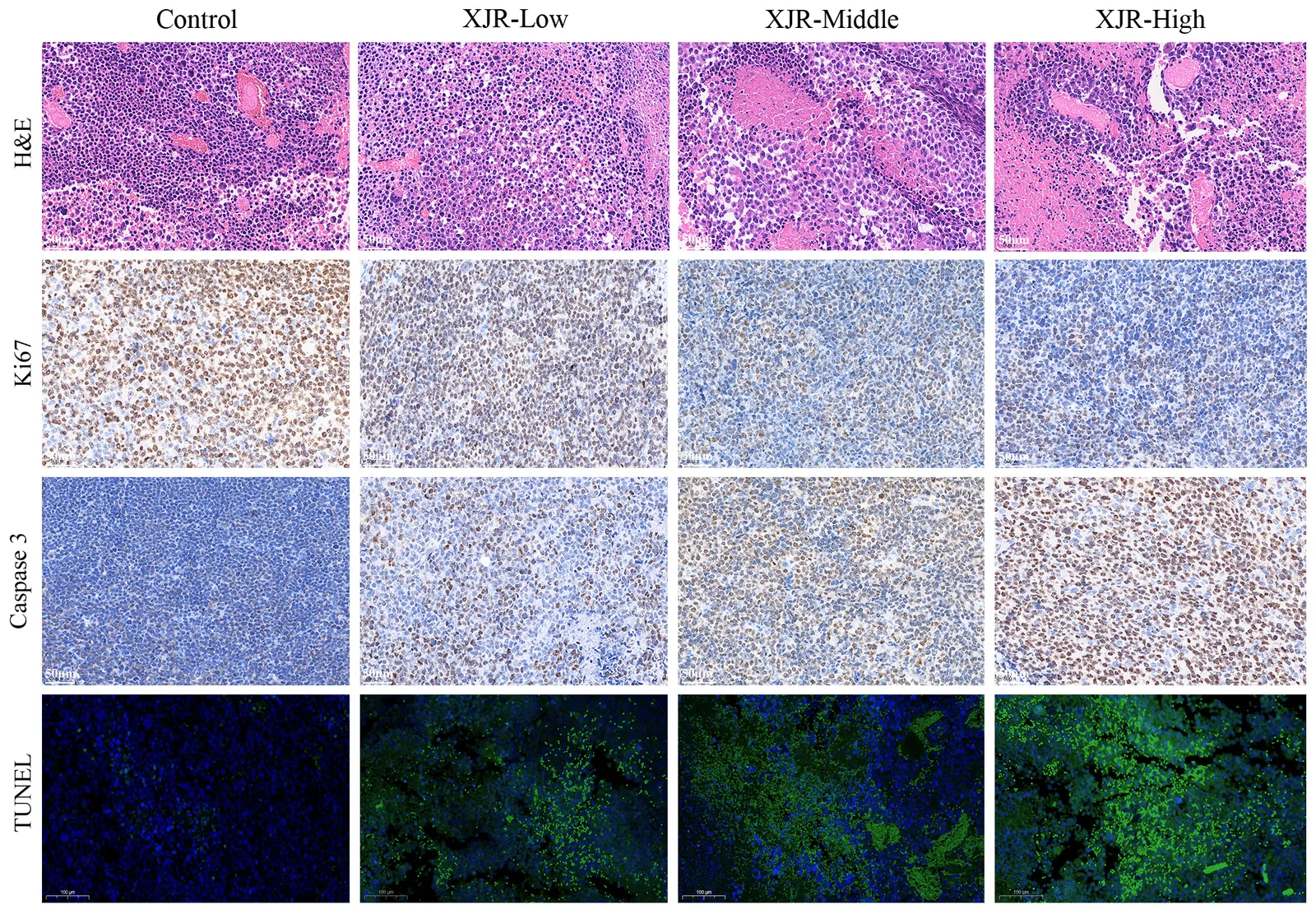
Inhibitory effect of XJR on tumor growth in HCC. Representative images of H&E staining, immunohistochemistry staining of Ki67, Caspase 3 and the TUNEL assay in tumor pathological sections of mice in each group. Magnification ×400.

### 3.2 XJR regulates inflammatory responses in HCC via promoting NLRP3 inflammasome activation

Induction of tumor cell death serves as an effective strategy to restrain HCC progression. Pyroptosis, an inflammatory form of programmed cell death typically triggered by pathogenic infection, is characterized by inflammasome activation and the release of pro-inflammatory mediators including IL-1β and IL-18 (27). Growing evidence has demonstrated that pyroptosis modulates the progression of hepatocellular carcinoma by regulating immune-related tumor cell death, thereby exerting an antitumor function (28, 29). Inflammatory cytokines including IL-1β and IL-18 have been employed to evaluate inflammatory state in serum of mice (**Figures 2A, B**) and HepG2 cells (**Figures 2C, D**). Compared with the Control group, the levels of pro-inflammatory cytokines were significantly upregulated both *in vivo* and *in vitro* following XJR treatment. Furthermore, LDH release assay for cytotoxicity assessment revealed that XJR induced elevated LDH release in HepG2 cells (**Figure 2E**), implying that XJR is capable of triggering pyroptosis in HCC. NLRP3 acts as a key intracellular sensor that governs the tightly controlled assembly of inflammasome complexes, thereby initiating downstream inflammatory responses through IL-1β and IL-18 and triggering pyroptosis. Given that these XJR-regulated cytokines represent major downstream effectors of NLRP3 signaling pathway, we speculate that the NLRP3 pathway exerts an essential function in the inflammatory response of HCC. Whether XJR modulates the NLRP3 inflammasome requires further investigation.

**Figure 2:**
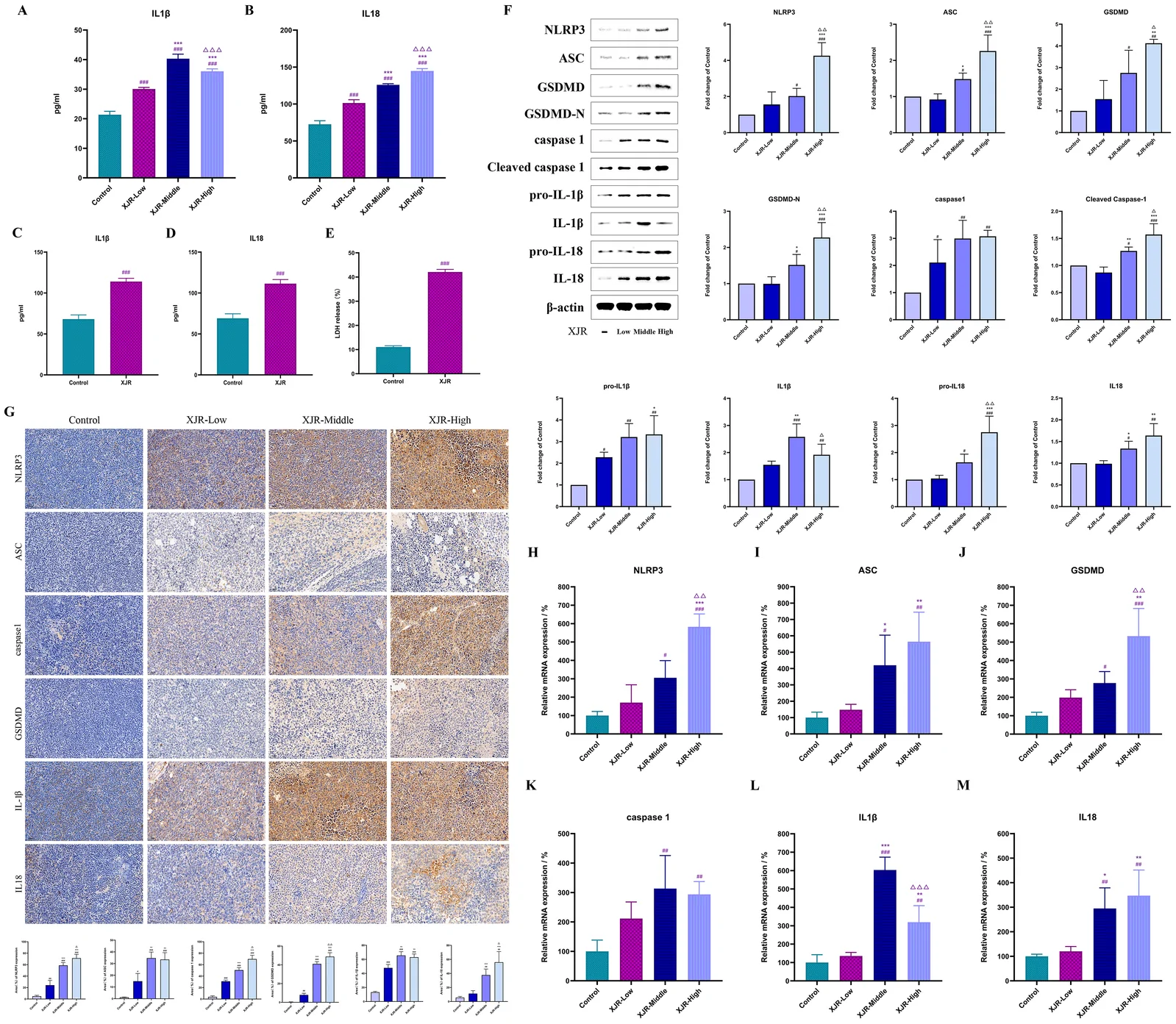
Effect of XJR on NLRP3-related pyroptosis and inflammatory responses in hepatocellular carcinoma. IL-1β and IL-18 levels of mice serum **(A, B)** and HepG2 cells **(C, D)** detected by ELISA. **(E)** The LDH release of HepG2 cells was tested by LDH assay (n=3). **(F)** The effect of XJR on the protein level and quantitative analysis of NLRP3, ASC, GSDMD, GSDMD-N, Caspase 1, Cleaved caspase 1, pro-IL-1β, IL-1β, pro-IL-18 and IL-18 in tumor tissue of each group (n=3). **(G)** Immunohistochemical staining assay of NLRP3, ASC, Caspase 1, GSDMD, IL-1β and IL-18 intensity in tumor tissue, Magnification ×200. **(H–M)** The effect of XJR on the relative messenger RNA level of *NLRP3*, *ASC*, *Caspase 1*, *GSDMD*, *IL-1β* and *IL-18* in tumor tissue of HCC mice (n=3). ^#^P < 0.05, ^##^P < 0.01 and ^###^P < 0.001 vs Control group; ^*^P < 0.05, ^**^P < 0.01 and ^***^P < 0.001 vs XJR-Low group; ^△^P < 0.05, ^△△^P <0.01 and ^△△△^P < 0.001 vs XJR-Middle group.

Moreover, western blot and IHC analyses revealed that the protein levels of key components within the NLRP3 signaling pathway were altered in tumor tissues. Specifically, the expression of the NLRP3 inflammasome and its downstream targets exhibited a dose-dependent increase following XJR treatment, indicating the regulatory effect of XJR on HCC (**Figures 2F, G**). Meanwhile, the mRNA levels measured by PCR were consistent with the above findings, further demonstrating that XJR upregulates the expression of the NLRP3 inflammasome and its downstream mediators (**Figures 2H–M**). Overall, compared with the Control group, XJR substantially promotes the NLRP3 signaling pathway at the transcriptional level, particularly in the high-dose XJR group. Therefore, we infer that XJR intervenes in the inflammatory response in HCC by upregulating NLRP3 signaling pathway.

### 3.3 XJR engaged in the upregulation of NLRP3 signaling pathway in HepG2 cells

As demonstrated in this study, XJR regulates NLRP3-associated pyroptosis and inflammatory responses *in vivo*. To further verify the precise function of XJR *in vitro*, MCC950, a potent and selective small-molecule inhibitor of the NLRP3 pathway (30), was used for subsequent experiments. Consistent with the above findings, apoptosis in XJR-treated HepG2 cells was assessed via the TUNEL assay. TUNEL signals were markedly increased in the XJR group, and this therapeutic effect was abrogated upon MCC950 intervention (**Figure 3A**). Meanwhile, the stimulatory effect of XJR on IL-1β and IL-18 expression in HepG2 cells was comparable to that observed *in vivo*; however, this effect was markedly attenuated by MCC950 (**Figures 3B, C**).

**Figure 3:**
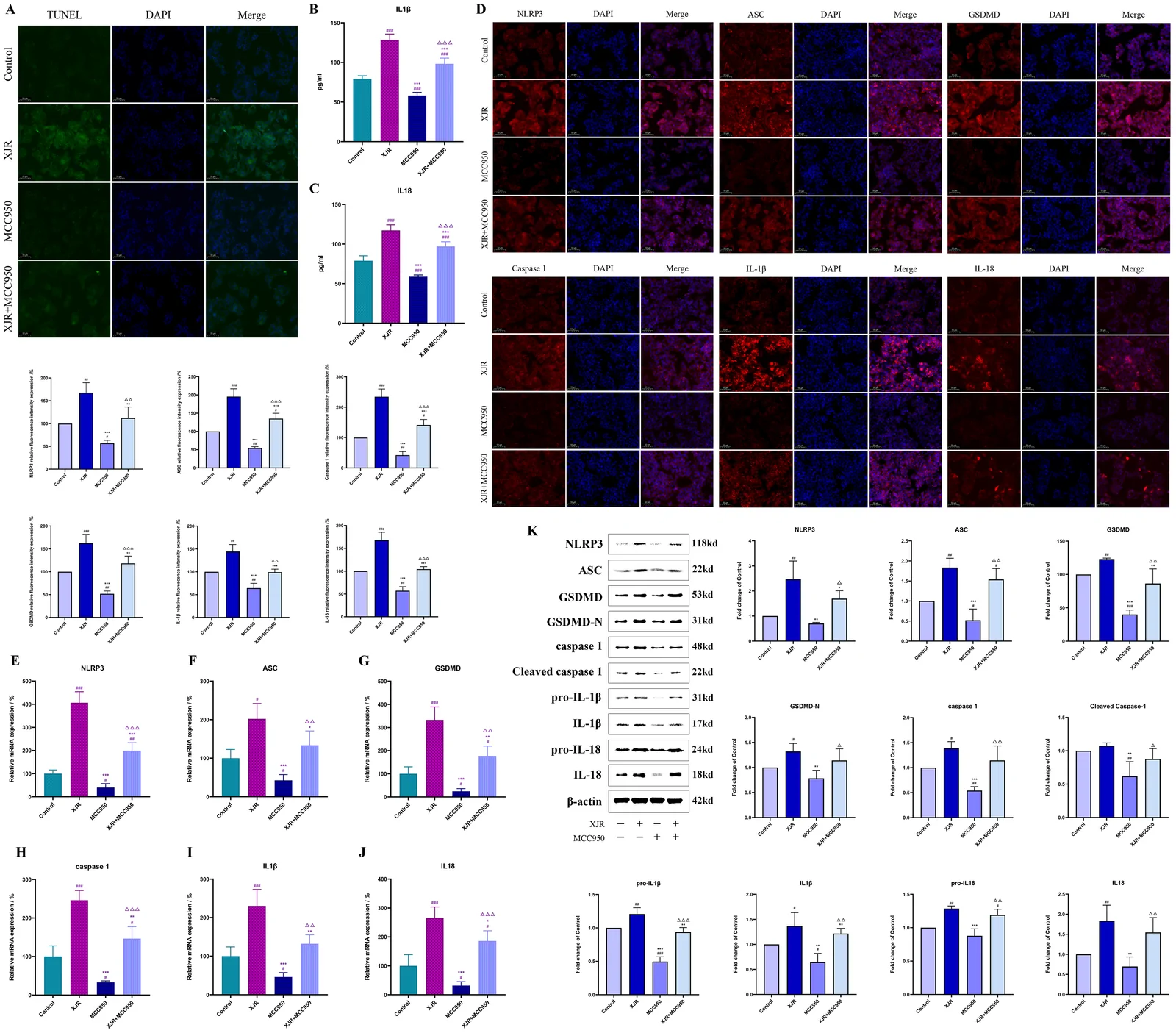
In hepatocellular carcinoma cells, the effect of XJR on NLRP3 signaling pathway and related downstream targets was reversed by MCC950. **(A)**TUNEL assay detects expression of apoptosis in HepG2 cells. **(B, C)** Levels of IL-1β and IL-18 in each group were tested by ELISA (n=3). **(D)** Immunofluorescence of NLRP3, ASC, Caspase 1, GSDMD, IL-1β and IL-18 in HepG2 cells. Magnification ×400. **(E–J)** The effect of XJR on the relative messenger RNA level of *NLRP3*, *ASC*, *Caspase 1*, *GSDMD*, *IL-1β* and *IL-18* in hepatocellular carcinoma cells (n=3). **(K)** The effect of XJR on the protein level and quantitative analysis of NLRP3, ASC, GSDMD, GSDMD-N, Caspase 1, Cleaved caspase 1, pro-IL-1β, IL-1β, pro-IL-18 and IL-18 in HepG2 cells of each group (n=3). ^#^P < 0.05, ^##^P < 0.01 and ^###^P < 0.001 vs Control group; ^*^P < 0.05, ^**^P < 0.01 and ^***^P < 0.001 vs XJR group; ^△^P < 0.05, ^△△^P <0.01, ^△△△^P < 0.001 vs MCC950 group.

Furthermore, addition of MCC950 inhibitor partially reversed the upregulation of XJR on NLRP3 signaling pathway in HepG2 cells according to the immunofluorescence staining (**Figure 3D**). Besides, mRNA (**Figures 3E–J**) and protein levels (**Figure 3K**) *in vitro* also exhibited a distinct efficacy analogous to that described above following XJR treatment. However, compared with the XJR-only group, co-treatment with MCC950 markedly reversed the upregulated expression of these key markers (NLRP3, ASC, Caspase-1, GSDMD, IL-1β, and IL-18) to different extents. These findings suggest that inhibition of the NLRP3 inflammasome attenuates the antitumor effects of XJR in HCC. Taken together, our results indicate that XJR can activate the NLRP3 inflammasome and its downstream signaling cascade, revealing that the activation of the NLRP3 inflammasome may contribute, at least in part, to the anti-HCC activity of XJR.

### 3.4 XJR suppressed mitophagy and induces mitochondrial dysfunction *in vivo*

To validate our hypothesis that the anti-HCC activity of XJR involves the PINK1/parkin signaling pathway, a panel of IHC analyses was performed on tumor tissues. Immunostaining revealed more PINK1 and parkin-positive cells in the Control group, whereas PINK1 and parkin expression decreased in a dose-dependent manner in XJR-treated tumor tissues, besides, p62 and LC3B in the XJR treatment groups emerged an upward trend than those in Control group (**Figure 4A**). Additionally, the Control group displayed minimal mitochondrial damage. In comparison, the low-dose XJR group exhibited mild mitochondrial swelling and degeneration; the outer mitochondrial membranes remained intact without rupture, whereas cristae structures showed partial, mild focal dissolution while most cristae were retained. A small number of vesicle-like lipid droplets were observed within the cells, along with a few vesicular structures extracellularly. Simultaneously, multiple sites of dissolution and rupture were observed in the outer nuclear membrane in the XJR-Middle and XJR-High group. Mitochondria exhibited moderate to severe edema and degeneration, with partial or extensive rupture of the outer membranes. Most cristae were dissolved, with only sparse remnants, accompanied by vacuolar degeneration. The mitochondrial matrix exhibited decreased electron density. Notably, rough endoplasmic reticulum was markedly dilated. Numerous lipid droplets were observed within the cells, alongside extensive rupture of the cell membrane and leakage of cytoplasmic contents. Abundant vesicular structures were detected both intracellularly and extracellularly, with focal discontinuities and perforations in the cell membrane (**Figure 4B**).

**Figure 4:**
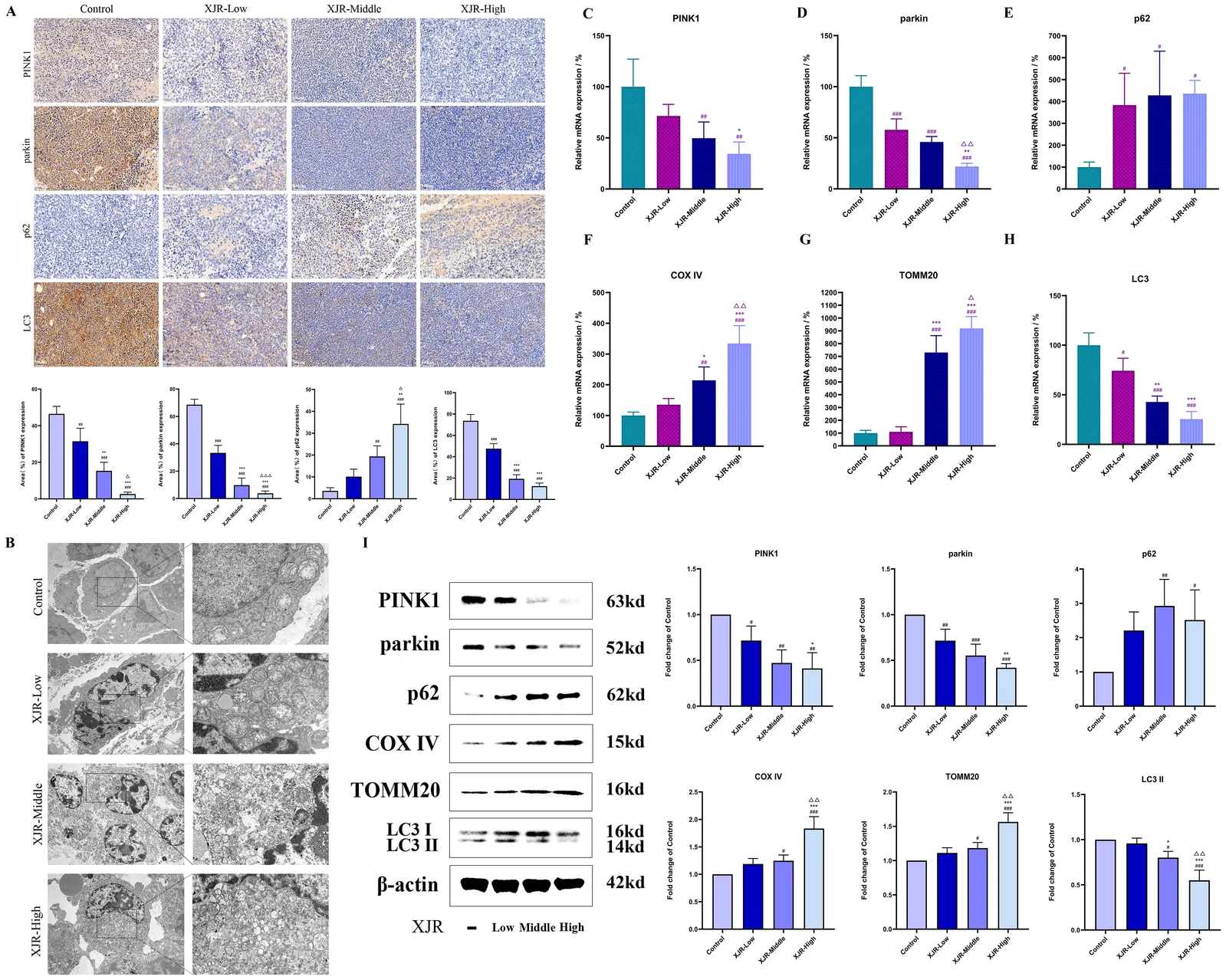
XJR modulates mitochondrial dysfunction and influences PINK1/parkin signaling pathway in tumor tissues of HCC. **(A)** Immunohistochemical staining of PINK1, parkin, p62 and LC3B intensity in tumor. Magnification×200. **(B)** Representative TEM images of mice tumor samples (magnification, 3000× and 10000×, respectively). **(C–H)** The effect of XJR on the relative messenger RNA level of *PINK1*, *parkin*, *p62*, *COX IV*, *TOMM20* and *LC3B* in tumor tissue of mice (n=3). **(I)** The effect of XJR on the protein level of PINK1, parkin, p62, COX IV, TOMM20 and LC3B with quantitative analysis in tumor of each group (n=3). ^#^P < 0.05, ^##^P < 0.01 and ^###^P < 0.001 vs Control group; ^*^P < 0.05, ^**^P < 0.01 and ^***^P < 0.001 vs XJR-Low group; ^△^P < 0.05, ^△△^P < 0.01 and ^△△△^P < 0.001 vs XJR-Middle group.

Consistent with IHC staining, RT-qPCR assays also demonstrated a significant reduction in the relative mRNA expression levels of *PINK1*, *parkin* and *LC3B*, as well as an upward trend in *p62*, *COX IV* and *TOMM20* with treatment of XJR especially in XJR-High group (**Figures 4C–H**). For the purpose of further elucidating the underlying mechanism by which XJR regulates the PINK1/parkin signaling pathway, the expression levels of key proteins PINK1, parkin, p62, COX IV, TOMM20 and LC3B in tumor tissue were determined (**Figure 4I**). Consistent protein-level changes were observed, and these findings collectively suggest that the anti-HCC effect of XJR may be associated with modulation of the PINK1/parkin signaling pathway.

### 3.5 XJR attenuated PINK1-mediated mitophagy and interfered with mitochondrial ROS in HepG2 cells

To further identify the functional target of XJR, 3-MA, a classic autophagy inhibitor, was used in subsequent experiments. As a widely adopted reagent in mitophagy studies, 3-MA blocks autophagosome formation. Analysis of key protein expression revealed that 3-MA reversed the inhibitory effects on mitochondrial damage, ROS generation, and NLRP3-associated inflammatory responses in HCC (31, 32). Similarly, the experiments *in vitro* further verified the intervention effect of XJR on apoptosis and mitoROS. The TUNEL assay exhibits the ascending expression in XJR group and shows the most distinctly improved therapeutic effect with addition of 3-MA (**Figure 5A**). Consistent with the apoptotic findings, fluorescence detection using DCFH-DA and MitoSOX demonstrated markedly elevated mitochondrial reactive oxygen species (mtROS) levels upon XJR treatment (**Figures 5B, C**). Compared with the Control group, the XJR group exhibited higher fluorescence intensity, indicating elevated intracellular oxidative stress (OS) levels following treatment. Meanwhile, combined treatment with XJR and 3-MA further intensified this phenotype.

**Figure 5:**
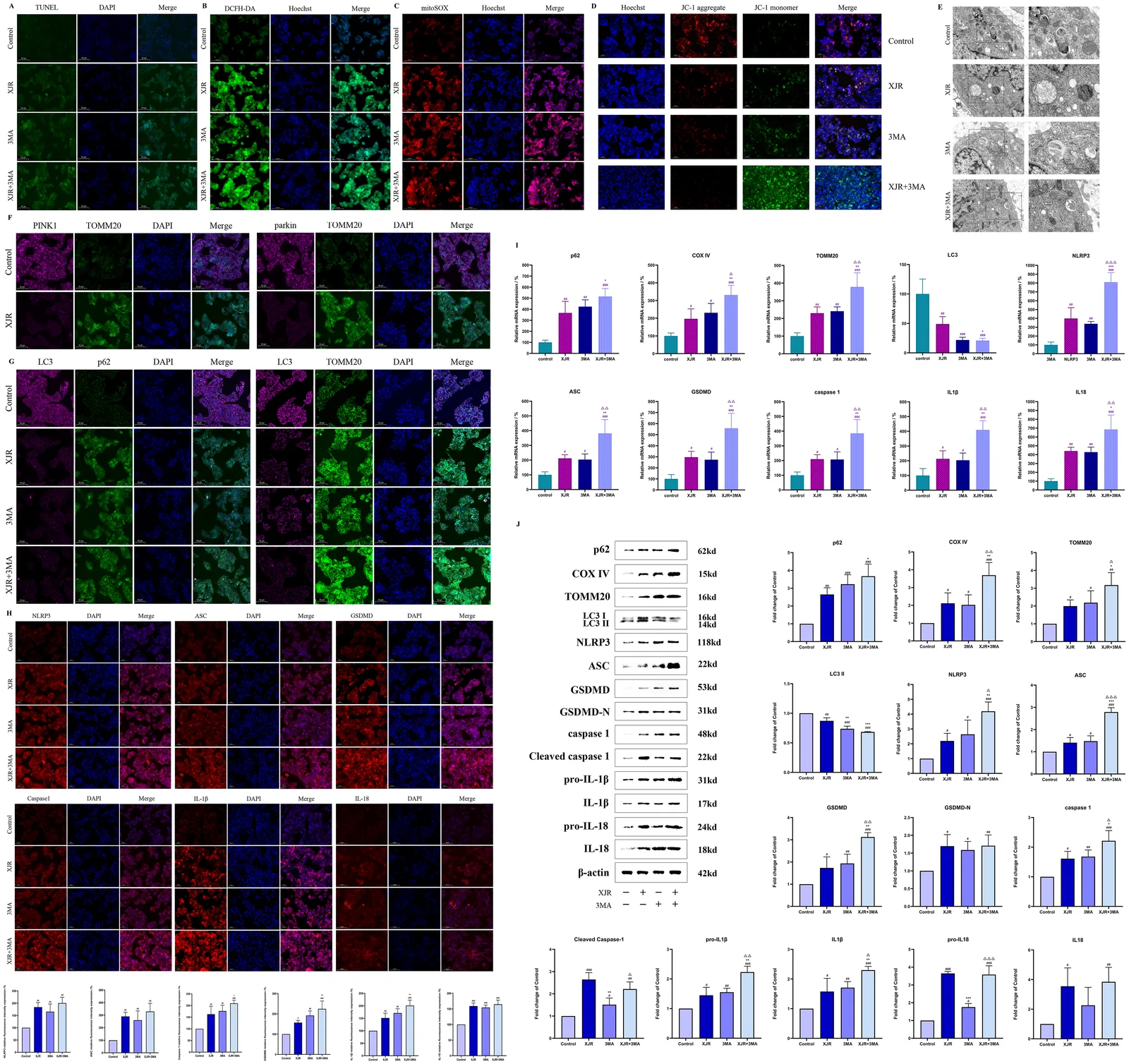
XJR alleviated mitophagy and attenuated expressions of PINK1/parkin signaling pathway in HepG2 cells. Representative images of **(A)** Immunofluorescence staining of TUNEL assay. Magnification ×400. **(B, C)** The production level of ROS in HepG2 cells was detected by means of the fluorescent probes DCFH-DA and mitoSOX; **(D)** The changes in MMP in HepG2 cells were measured using the JC-1 kit. The magnification was set at ×20. **(E)** Autophagosomes and autolysosomes in HepG2 cells, following a 24-hour incubation with XJR, were observed via TEM. (magnification, 3000× and 10000×, respectively). **(F–H)** Immunofluorescence staining of PINK1/parkin-mitophagy and NLRP3 signaling pathway. Magnification ×400. **(I)** The effects of XJR on the relative messenger RNA level of *p62*, *COX IV*, *TOMM20*, *LC3B*, *NLRP3*, *ASC*, *GSDMD*, *caspase 1*, *IL-1β* and *IL-18 in vitro* (n=3). **(J)** The effects of XJR on the protein level of p62, COX IV, TOMM20, LC3B, NLRP3, ASC, GSDMD, GSDMD-N, Caspase 1, Cleaved caspase 1, pro-IL-1β, IL-1β, pro-IL-18 and IL-18 in HepG2 cells in each group (n=3). ^#^P < 0.05, ^##^P < 0.01 and ^###^P < 0.001 vs Control group; ^*^P < 0.05, ^**^P < 0.01 and ^***^P < 0.001 vs XJR group; ^△^P < 0.05, ^△△^P <0.01 and ^△△△^P < 0.001 vs 3-MA group.

To further explore how XJR effect on HepG2 cells, intracellular mitochondria were observed (**Figure 5D**). Reduced mitochondrial membrane potential (MMP), detected via the JC-1 assay, serves as a key marker of mitochondrial dysfunction. XJR-treated HepG2 cells displayed increased green fluorescence and diminished red fluorescence intensity, indicating decreased MMP in the treatment group. Furthermore, upon co-intervention with XJR and 3-MA, red fluorescence intensity was further diminished, indicating that XJR combined with 3-MA exerted a synergistic effect on MMP loss, consistent with our expectation. According to the performance of the TEM, mitochondria exhibited mild edema and degeneration, with numerous autophagolysosomes and autophagosome structures were visible in the Control group. In the XJR group, the nuclei displayed irregular morphology with convoluted shapes and multiple surface depressions. Some mitochondria exhibited severe edema and degeneration, with extensive rupture of the outer membrane. Most cristae were dissolved, with few remnants, presenting vacuolar degeneration. Few autophagolysosomes were observed, and no prominent autophagosome structures were detected. Contemporary, nuclei exhibited marked morphological irregularities, with multiple nuclear clefts and a gyriform appearance upon combined XJR and 3-MA treatment. Meanwhile, mitochondria displayed markedly reduced volume, with regions of the mitochondrial matrix showing elevated electron density. The outer membranes and cristae presented pyknotic alterations, and no obvious autophagolysosomes or autophagosomes were detected (**Figure 5E**). These results showed that XJR can act on the mitochondrial function in HCC, which was related to oxidative stress levels and MMP in cells.

As depicted in **Figure 5F**, after treated with XJR, the fluorescence intensity of PINK1 and parkin were restrained, followed by the evident of the mitophagy targets and activated NLRP3 that were significantly enhanced by XJR-containing serum treatment (**Figures 5G, H**). Also, mRNA and protein expression data (**Figures 5I, J**) demonstrated that XJR upregulated key targets of mitophagy and the NLRP3 signaling pathway, consistent with the TEM observations. As XJR exhibited an equal improvement effect with 3-MA, indicating the facilitator role of XJR in NLRP3 inflammasome and the downstream signaling pathway, as well as a mitophagy-inhibitor-efficacy on HCC. Collectively, XJR exerted an anti-mitophagy effect analogous to 3-MA, that is, both agents upregulated the NLRP3 inflammasome and its downstream targets. Furthermore, we infer that XJR may specifically suppress mitophagy, thereby accelerating NLRP3 inflammasome-associated pyroptosis and inflammatory responses in HCC.

### 3.6 Suppression of PINK1-directed mitophagy involved preventive effects of XJR *in vitro*

Given that PINK1 has been confirmed to be a critical regulatory factor in mitophagy, we performed further experiments to clarify its mediating role in XJR-induced NLRP3 inflammasome activation and mitochondrial dysfunction in HepG2 cells. To this end, PINK1-specific siRNA was utilized to downregulate the endogenous PINK1 expression in HepG2 cells. We verified the knockdown efficiency of PINK1-siRNA at mRNA and protein levels. Both qRT-PCR and Western blot data revealed significantly decreased PINK1 expression following PINK1-siRNA transfection relative to the negative control group, which validated the effective interference of PINK1. TUNEL, DCFH-DA and MitoSOX assays revealed elevated signals following siPINK1 transfection or XJR treatment, reflecting increased apoptosis and ROS accumulation. As anticipated, the combination of siPINK1 and XJR further enhanced these pro-tumoral effects (**Figures 6A–C**).

**Figure 6:**
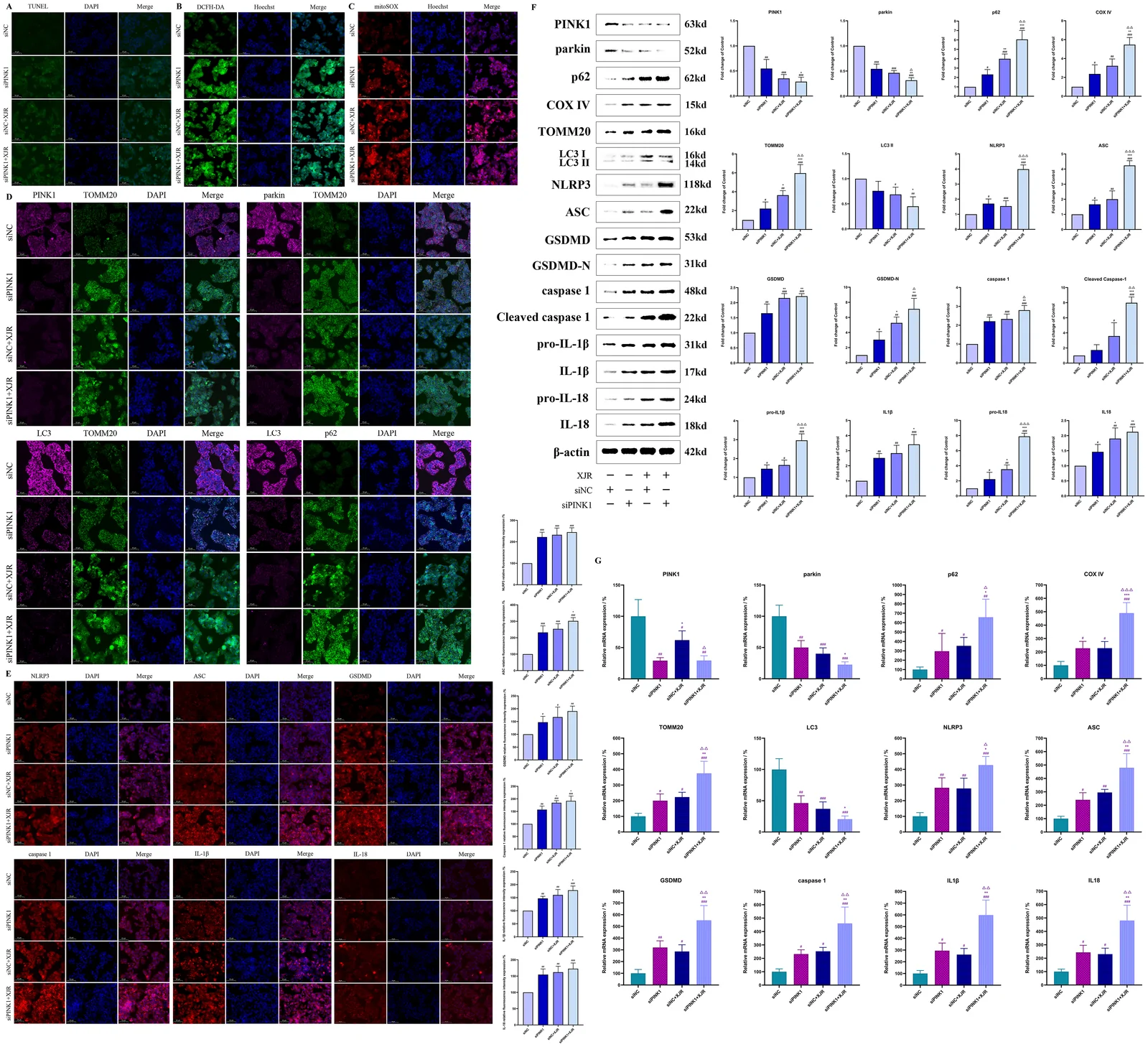
Effect of NLRP3 inflammasome activation and mitochondrial dysfunction by XJR is mediated by PINK1 in HepG2 cells. Cells were transduced with NC-siRNA or PINK1-siRNA, and then pretreated with or without XJR. Representative images of **(A)** Immunofluorescence staining of TUNEL assay Magnification ×400. **(B, C)** the level of ROS production in HepG2 cells by DCFH-DA and mitoSOX; **(D, E)** Immunofluorescence staining of PINK1/parkin-mitophagy and NLRP3 signaling pathway, Magnification ×400. **(F)** The effects of XJR on the protein level of p62, COX IV, TOMM20, LC3B, NLRP3, ASC, GSDMD, GSDMD-N, Caspase 1, Cleaved caspase 1, pro-IL-1β, IL-1β, pro-IL-18 and IL-18 in HepG2 cells in each group (n=3); **(G)** The effects of XJR on the relative messenger RNA level of *p62*, *COX IV*, *TOMM20*, *LC3B*, *NLRP3*, *ASC*, *GSDMD*, *caspase 1*, *IL-1β* and *IL-18 in vitro* (n=3). ^#^P < 0.05, ^##^P < 0.01 and ^###^P < 0.001 vs siNC group; ^*^P < 0.05, ^**^P < 0.01 and ^***^P < 0.001 vs siPINK1 group; ^△^P < 0.05, ^△△^P <0.01 and ^△△△^P < 0.001 vs siNC+XJR group.

XJR suppressed mitophagy and induced mitochondrial network fragmentation. To further verify whether mitophagy inhibition synergistically potentiates the antitumor effects of XJR, immunofluorescence staining was conducted to assess the PINK1/parkin mitophagy axis and NLRP3 signaling pathway. The results showed reduced fluorescence intensity of PINK1, parkin and LC3B, along with decreased colocalization of these proteins with TOMM20. In contrast, the expression of p62, NLRP3, ASC, GSDMD, caspase-1, IL-1β and IL-18 was markedly elevated in the siPINK1-transfected or XJR-treated groups. Moreover, combined treatment produced a synergistic effect, indicating that XJR exerts its function through regulation of PINK1-mediated mitophagy and NLRP3 inflammasome (**Figures 6D, E**). Meanwhile, as shown in **Figures 6F, G**, the cellular protein level and mRNA expression of PINK1/parkin-mitophagy and NLRP3 signaling pathway relative markers in HCC cells were determined, which revealed the downregulation of mitophagy after exposed to XJR and which subsequently facilitate NLRP3, ASC, GSDMD, GSDMD-N, Caspase 1, Cleaved caspase 1, pro-IL-1β, IL-1β, pro-IL-18 and IL-18 expression to varying degrees. The proportion of damaged mitochondria and NLRP3 activation in siPINK1-treatment or XJR group was increased and emerged a synergistic effect, with lower mitophagy level than that in the Control group. Collectively, these observations imply that the PINK1/parkin/NLRP3 signaling axis might contribute to the biological responses induced by XJR.

## 4 Discussion

How to block or reverse HCC is remaining difficult clinical subjects nowadays. TCM has raised increasing attention with a unique role in HCC treatment (17). With long-term clinical application, XJR is a TCM formula with favorable clinical safety profiles. It effectively relieves symptoms and improves clinical conditions in patients with HCC, achieving promising therapeutic effects. Our previous work has performed multiple mechanistic investigations into XJR. The present study further dissects the therapeutic mechanisms of XJR against HCC using cellular and animal models, focusing on its regulation of mitophagy and NLRP3 inflammasome activation (**Figure 7**).

**Figure 7:**
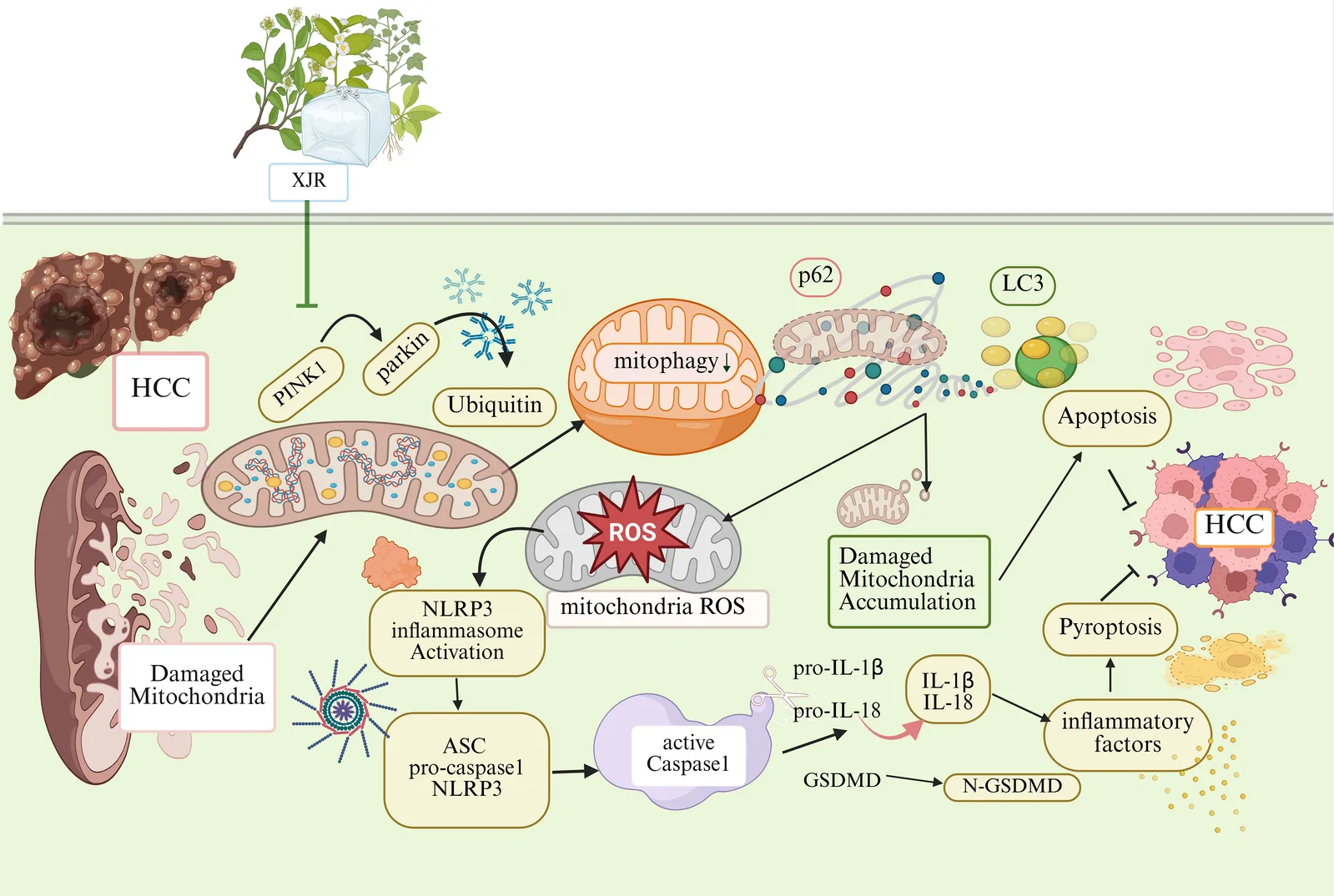
Schematic diagram of the potential mechanisms underlying the protective effects of XJR on HCC. XJR acts a protective role in HCC and exerts an alleviation on PINK1-mediated mitophagy. Meanwhile, XJR induces activation of the NLRP3 inflammasome and facilitates ASC and caspase 1, resulting in increasing maturation and secretion of IL-1β and IL-18 remarkedly. XJR promotes NLRP3 signaling pathway by suppressing PINK1-directed mitophagy, thus preventing the progression of HCC.

Mitophagy describes the selective autophagic turnover of damaged mitochondria: depolarized mitochondria are sequestered inside double-membraned autophagosomes and subsequently delivered to lysosomes for degradation. This process exerts pivotal functions in governing cancer metabolic reprogramming, sustaining cellular homeostasis, maintaining cancer stemness, and conferring chemoradiotherapy resistance, all of which facilitate tumor cell adaptation to the tumor microenvironment (TME) (33). The TME is a major regulator of mitochondrial dysfunction and inflammatory signaling in HCC. Recent studies demonstrate that metabolically reprogrammed tumor-associated macrophages (TAMs) induce mitochondrial stress, excessive ROS generation, and inflammatory activation in hepatic tumor cells through paracrine crosstalk (34–37). Although our present investigation centers on the intrinsic PINK1/parkin mitophagy-NLRP3 signaling cascade operating within HCC cells, these TAM-related observations provide a broader TME framework for our model. Crosstalk between tumor cells and metabolically rewired TAMs may further modulate mitophagic activity and NLRP3 inflammasome activation, representing an important direction for future exploration of this signaling axis.

Mitophagy is of crucial significance in maintaining mitochondrial quality, and the PINK1/parkin pathway stands as the most extensively researched mechanism underlying this process. When mitochondrial damage occurs or membrane potential is lost, PINK1 accumulates on the outer mitochondrial membrane, where it further activates the E3 ubiquitin ligase function of parkin. This results in the ubiquitination of mitochondrial surface proteins, recruiting autophagy adaptors such as p62/SQSTM1, which then targets damaged mitochondria for engulfment by autophagosomes. Ultimately, these mitochondria are degraded through fusion with lysosomes (38). PINK1-dependent mitochondrial quality control represents a critical biological axis governing tumor cell fate. Recent studies of gastric adenocarcinoma demonstrate that PINK1 insufficiency confers specific mitochondrial vulnerability on cancer cells (39, 40). Although those investigations were performed in gastric cancer rather than HCC, these reports support the broader notion that the functional status of PINK1-mediated mitochondrial quality control dictates tumor cell sensitivity to mitochondrial stress. This complementary evidence contextualizes our observations in HCC cells and strengthens the translational relevance of our findings connecting XJR treatment, PINK1/parkin mitophagy, and anti-tumor responses.

Mitophagy can maintain mitochondrial quality and quantity in cells, thereby preserving cellular homeostasis and promoting the survival of HCC cells under stress conditions (41). In early-stage HCC, mitophagy plays a protective role by clearing damaged mitochondria, attenuating ROS generation, preserving intracellular redox homeostasis, and mitigating cellular stress and genomic damage (42). Mitophagy sustains mitochondrial membrane potential, alleviates mitochondrial damage, and reduces intracellular ROS generation. This pathway mitigates oxidative stress induced by chemotherapeutic agents, enabling HCC cells to adapt to the therapeutic microenvironment and ultimately facilitating chemoresistance (43). Moreover, 3-MA, which blocks the fusion between autophagosomes and lysosomes, can synergize with cisplatin, doxorubicin, and sorafenib to strengthen their antitumor effects against HCC (44). Similarly, autophagy suppression via 3-MA or inactivation of ATg4B inhibits the proliferation of Huh7 cells (45). It should be noted that 3-MA serves as a broad-spectrum autophagy inhibitor rather than a selective mitophagy inhibitor. Therefore, phenotypic alterations observed upon 3-MA pretreatment cannot be fully attributed to PINK1/parkin-mediated mitophagy. This non-specificity represents a limitation of the present study. For this reason, we cautiously conclude that the rescue effects observed following 3-MA pretreatment preliminarily suggest the involvement of autophagy and mitophagy in XJR-mediated anti-HCC activity. These findings should not be overinterpreted as definitive evidence for mitophagy-specific regulation. Future genetic manipulation of mitophagy-specific regulators will be required to precisely delineate the distinct contribution of mitophagy within this regulatory network.

Besides, suppression of PINK1-parkin-mediated mitophagy results in the release of mitochondrial DNA into the cytoplasm, and this process in turn triggers the generation of inflammatory cytokines. The combined actions of mitophagy inhibition and inflammatory activation work together to suppress the proliferation of HCC cells (46). Available evidence indicates that the PINK1/parkin signaling pathway governs mitophagy, thereby clearing damaged mitochondria, curbing excessive ROS generation, suppressing NLRP3 inflammasome activation, and promoting mitochondrial repair (47–49). Composed of NLRP3, the adaptor protein ASC, and caspase-1, the NLRP3 inflammasome functions as a critical multiprotein complex, which is responsible for mediating the proteolytic activation of caspase-1 (50). Impaired mitophagy leads to oxidative stress and the accumulation of damaged mitochondria. These changes further trigger assembly and activation of the NLRP3 inflammasome, promote ASC oligomerization and caspase-1 cleavage, and ultimately increase the secretion of proinflammatory cytokines such as IL-1β and IL-18 (51). Accumulating evidence indicates that NLRP3 protein expression is downregulated in the majority of hepatocellular carcinoma cells, accompanied by decreased levels of ASC and pro-caspase-1, which are essential components of the NLRP3 inflammasome. Notably, NLRP3 inflammasome activation has been reported to restrain DNA replication and cell proliferation in HCC cells. Moreover, this activation also suppresses the proliferative potential of HCC cells. Consequently, the tumor-suppressive effect of NLRP3 inflammasome activation in HCC may be achieved through the inhibition of cell proliferation and migration (52).

The NLRP3 inflammasome exerts context-dependent dual functions in HCC instead of uniformly pro- or anti-tumor activity, with its phenotype governed by tumor stage, hepatic microenvironment (inflammation, fibrosis, metabolic stress), and cell-type-specific activation in malignant and stromal immune cells. At pre-tumorigenic and early HCC stages, moderate NLRP3 activation acts as a tumor-suppressive barrier: hepatic injury, oxidative stress, and viral/metabolic inflammation trigger caspase-1-dependent pyroptosis to clear damaged hepatocytes and premalignant clones, limiting oncogenic chronic inflammation. Functional NLRP3 signaling mitigates liver fibrosis and inflammatory disturbance, restraining HCC initiation and early growth. In established advanced HCC, by contrast, sustained excessive NLRP3 activation drives tumor-promoting effects. Aberrant NLRP3 signaling in tumor-associated macrophages and hepatic stellate cells induces proinflammatory cytokine release, remodeling the microenvironment into an immunosuppressive, proliferative, and pro-metastatic niche. This cascade facilitates HCC proliferation, epithelial-mesenchymal transition, angiogenesis and metastasis, dampens anti-tumor immunity, and accelerates disease progression and chemoresistance. In summary, NLRP3 function in HCC is stage-dependent: it suppresses early malignant transformation but promotes progression and immune escape in late-stage tumors. This duality accounts for conflicting observations in prior literature. Therapeutic targeting of the NLRP3 inflammasome for HCC thus demands stage-specific fine-tuning rather than indiscriminate activation or inhibition to maximize efficacy.

A close relationship has been observed between inflammation and tumorigenesis (53). Over the past decade, substantial progress has been achieved in delineating the mechanisms underlying inflammasome activation, its roles in HCC pathogenesis, and the intricate crosstalk between inflammasome signaling and other innate immune pathways (54). Pyroptosis, a form of programmed cell death, can exert antitumor effects and thus benefit the organism (55, 56). Accumulating evidence confirms that enhanced pyroptosis suppresses HCC progression. In contrast, attenuated pyroptosis facilitates HCC pathogenesis, largely through repression of the NLRP3 inflammasome. The activation of caspase-1-dependent pyroptosis can induce HCC cell death and effectively suppress the migration and proliferation of HCC cells (57, 58). These findings suggest that NLRP3-mediated pyroptosis represents a promising strategy for anticancer therapy. TUNEL staining fails to differentiate pyroptosis from apoptosis because it detects DNA damage originating from diverse cell death modalities. We evaluated both mRNA and protein levels of caspase-1 and GSDMD in this work. Identification of cleaved GSDMD-N and activated caspase-1 serves as core molecular evidence for pyroptotic activation. Though our data demonstrate pyroptosis induction, further pharmacological or genetic inhibition of caspase-1 or GSDMD is needed to verify causality in future studies. Mounting studies have demonstrated that NLRP3 inflammasome signaling plays context-dependent dual roles in the initiation and progression of HCC, which largely relies on hepatic pathological stages, cellular origin, tumor microenvironment and therapeutic interventions. During the premalignant transition from chronic hepatitis to liver cirrhosis, persistent NLRP3 inflammasome activation drives chronic hepatic inflammation via secretion of mature IL-1β and IL-18, remodels the immunosuppressive microenvironment, and accelerates the progression of hepatic fibrosis toward malignant transformation, thereby conferring potent pro-carcinogenic effects. Nevertheless, NLRP3 expression is commonly suppressed in established HCC tumor tissues, and loss of NLRP3 inflammasome activity is closely correlated with poorly differentiated histology and unfavorable clinical prognosis. In this context, targeted activation of NLRP3 signaling can induce pyroptotic cell death in HCC cells and restrict tumor proliferation and invasion. This renders the pathway tumor-suppressive and supports our present conclusion that XJR blocks HCC progression by promoting NLRP3 inflammasome activation and the subsequent pyroptosis. The pathological progression of HCC is closely associated with ROS, the NLRP3 inflammasome, and its downstream inflammatory mediators. A promising strategy for the prevention and treatment of HCC may lie in targeting PINK1/parkin-mediated mitophagy and the subsequent activation of the NLRP3 inflammasome. Collectively, these findings demonstrate that mitophagy inhibition promotes HCC cell death, and TCM exhibits unique advantages in HCC therapy. Accumulating evidence confirms that TCM can effectively suppress the proliferation and migration of HCC cells via targeted regulation of mitophagy (19). These research outcomes provide a robust scientific basis for the clinical application of TCM in cancer therapy.

The mechanistic bridge linking impaired mitophagy to NLRP3 inflammasome activation involves multiple mitochondrial damage-associated molecular patterns (mtDAMPs) (59). Candidate triggers include excessive mtROS accumulation, leakage of oxidized mtDNA into the cytosol, dissipation of mitochondrial membrane potential, and externalization of cardiolipin, all of which can initiate or amplify NLRP3 inflammasome assembly (60). These signals are not mutually exclusive and may function within a synergistic feedforward loop. Our findings indicate that XJR suppresses PINK1/parkin-dependent mitophagy, resulting in the accumulation of damaged mitochondria and subsequent NLRP3 inflammasome activation. Nevertheless, the present study cannot identify which mtDAMP acts as the primary upstream trigger. In view of the complexity of the mitophagy-NLRP3 signaling network described in existing reports, additional targeted experiments are needed to dissect the relative contribution of each mitochondrial danger signal in HCC cells following XJR intervention.

The present study indicated that XJR could mitigate PINK1/parkin-mediated mitophagy and then facilitate the oligomerization of ASC, NLRP3 inflammasome activation and assembly and then promote the subsequent caspase-1 activation and production of inflammatory cytokines. Characterized by multi-component and multi-target features, TCM can induce mitophagy through multiple pathways, thereby exerting anticancer effects and improving drug sensitivity. Meanwhile, TCM has been demonstrated to have a favorable safety profile with relatively low toxicity and fewer adverse reactions. Notably, XJR regulates apoptosis, pyroptosis and mitophagy in HCC, displaying multi-target selective activity. Further investigations are required to clarify whether XJR modulates the crosstalk between mitophagy and inflammatory responses via these multi-target mechanisms. Taken together, unraveling the connection of PINK1-mediated mitophagy and inflammasome activation will be contributing to instructing the anti-HCC activity and the further specific mechanism will further be discussed in subsequent future study. Although we have validated the pivotal role of PINK1/parkin-dependent mitophagy through 3-MA pharmacological blockade and PINK1 siRNA-mediated genetic knockdown, further overexpression rescue assays targeting PINK1 or parkin would strengthen the reliability of our conclusions. Although our data demonstrate that XJR treatment reduces PINK1/parkin signaling and elevates NLRP3 inflammasome activation, these findings currently support correlative rather than causal associations. Further rescue experiments, such as PINK1 or Parkin overexpression, will be required in future investigations to verify whether the regulation of NLRP3 by XJR is directly dependent on PINK1/parkin-mediated mitophagy. Also, we recognize that detection of mitophagic flux using mt-Keima or mito-QC would offer more direct evidence for altered mitophagy. This study focuses on delineating the crosstalk between mitophagy and the NLRP3 inflammasome upon XJR treatment. While we performed a preliminary assessment of mitophagy, we did not carry out in-depth characterization of mitophagic flux. Dedicated mitophagic flux assays will be conducted in our follow-up investigations. It should be noted that the *in vitro* experiments of this study were performed primarily in HepG2 cells, while the *in vivo* validation relied on a single H22 subcutaneous xenograft model. Meanwhile, although our current findings are reliable using this well-characterized HCC cell model and conserved tumor signaling pathways widely documented in the literature, further validation across additional HCC cell lines and clinical specimens remains necessary to consolidate the translational potential of XJR. Accordingly, we plan to perform multi-cell-line validation and verification using clinical tumor samples in our future systematic studies to further confirm and extend the generalizability of our observations. A thorough grasp of the pathogenesis of HCC, the identification of potential therapeutic targets, and the optimization of treatment strategies are of pivotal significance for the clinical management of the disease.

## 5 Conclusion

In general, our findings reveal that XJR activates the NLRP3 inflammasome via suppression of PINK1-dependent mitophagy, thereby restraining HCC progression. This study offers new mechanistic insights into the anti-HCC effects of XJR. Further investigations are warranted to dissect the interplay among XJR, NLRP3-associated mitophagy, oxidative stress, and pyroptosis.

## Data Availability

The original contributions presented in the study are included in the article/**Supplementary Material**. Further inquiries can be directed to the corresponding authors.

## Supplementary material

Supplementary Figure 1 Total ion flow chromatogram of positive and negative ions.

Supplementary Figure 2 Results of chemical composition analysis in XJR.

Supplementary Figure 3 Effect of XJR on body weight, tumor size and tumor weight in HCC xenograft mice
